# CMR endpoints for clinical trials: impact of operator experience on the accuracy of image analysis

**DOI:** 10.1186/1532-429X-14-S1-P38

**Published:** 2012-02-01

**Authors:** Elisa McAlindon, Jessica Harris, Helen Mathias, Debbie Marsden, Nathan Manghat, Barney Reeves, Chiara Bucciarelli-Ducci

**Affiliations:** 1 Bristol Heart Institute, NIHR Cardiovascular BRU, Bristol, UK

## Background

Cardiac magnetic resonance (CMR) imaging studies are increasingly being used as surrogate endpoints in clinical trials. There have been no previous studies investigating what level of training is required to provide an accurate and robust analysis of these parameters.

## Methods

20 CMR studies of acute coronary syndromes were included in this study. 6 observers with three levels of training conducted the image analysis: Observers 1+4: expert SCMR level 3 operators; Observers 2+5: trainees with SCMR level 2 experience; Observers 3+6: a cardiologist and a clinical trial coordinator with no previous CMR experience. The latter level underwent a 4hr tutorial on how to use the software on 10 practice cases. All 6 observers analysed 20 studies, and re-analysed 10 studies 24 hours after. Volumes and mass were analysed using semi-automated software (Argus, Siemens), infarct size (IS) was manually planimetered using a 2SD threshold.

## Results

Intra-observer variability was assessed using intraclass correlation coefficient (ICC), inter-observer variability was assessed using Bland Altman plots for agreement. Intra-observer variability was low for volumes, and was highest for mass measurements. (Representative results ICC; observer 1 EDV: 0.97, ESV: 0.99, mass: 0.95, IS: 0.99 vs observer 2 EDV: 0.97, ESV: 0.98, mass: 0.91, IS: 0.98 vs observer 3 EDV: 1.0, ESV: 1.0, mass: 0.91, IS: 0.93). When compared with the most experienced observer, inter-observer variability was highest for IS and increased with decreasing level of experience (Table 1 for representative results for 3 of the 6 observers at 3 differing levels of experience). Bland Altman plots suggested acceptable agreement. Full results for all 6 observers will be presented.

**Table 1:** Inter-observer variability

Observer		EDV* (ml)	ESV (ml)	Mass (g)	IS* (g)
1 vs 2	Mean (SD) difference	6.51 (8.92)	0.04 (0.13)	11.38 (15.08)	-0.15 (0.49)
1 vs 2	Mean (SD)	150.59 (30.29)	4.20 (0.40)	128.48 (26.95)	2.86 (1.06)
1 vs 2	Coefficient of variation (%)	5.92	3.04	11.74	17.04
1 vs 3	Mean (SD) difference	29.70 (15.83)	0.26 (0.23)	43.47 (26.81)	-0.74 (0.58)
1 vs 3	Mean (SD)	160.95 (36.28)	4.31 (0.41)	145.62 (35.55)	2.65 (1.01)
1 vs 3	Coefficient of variation (%)	9.84	5.29	18.41	21.75

Summary statistics calculated are mean and SD of differences, mean and SD of values. Differences between observers are assessed using Bland-Altman plots. *log-transformed for skewed distribution.

## Conclusions

Operator experience highly influences the accuracy of calculating CMR parameters. When CMR LV volumes and infarct size are used as surrogate endpoints for clinical trials, a SCMR level 3 observer is the recommended operator to perform the analysis. Based on the results of our study, a SCMR level 2 operator will require additional experience, and a non-experienced operator significant additional training.

## Funding

NIHR Cardiovascular Biomedical Research Unit.

